# Quantitative photoacoustic tomography: modeling and inverse problems

**DOI:** 10.1117/1.JBO.29.S1.S11509

**Published:** 2023-12-20

**Authors:** Tanja Tarvainen, Ben Cox

**Affiliations:** a University of Eastern Finland, Department of Technical Physics, Kuopio, Finland; b University College London, Department of Medical Physics and Biomedical Engineering, London, United Kingdom

**Keywords:** photoacoustic imaging, tomography, inverse problems, quantitative imaging, radiative transfer

## Abstract

**Significance:**

Quantitative photoacoustic tomography (QPAT) exploits the photoacoustic effect with the aim of estimating images of clinically relevant quantities related to the tissue’s optical absorption. The technique has two aspects: an acoustic part, where the initial acoustic pressure distribution is estimated from measured photoacoustic time-series, and an optical part, where the distributions of the optical parameters are estimated from the initial pressure.

**Aim:**

Our study is focused on the optical part. In particular, computational modeling of light propagation (forward problem) and numerical solution methodologies of the image reconstruction (inverse problem) are discussed.

**Approach:**

The commonly used mathematical models of how light and sound propagate in biological tissue are reviewed. A short overview of how the acoustic inverse problem is usually treated is given. The optical inverse problem and methods for its solution are reviewed. In addition, some limitations of real-life measurements and their effect on the inverse problems are discussed.

**Results:**

An overview of QPAT with a focus on the optical part was given. Computational modeling and inverse problems of QPAT were addressed, and some key challenges were discussed. Furthermore, the developments for tackling these problems were reviewed. Although modeling of light transport is well-understood and there is a well-developed framework of inverse mathematics for approaching the inverse problem of QPAT, there are still challenges in taking these methodologies to practice.

**Conclusions:**

Modeling and inverse problems of QPAT together were discussed. The scope was limited to the optical part, and the acoustic aspects were discussed only to the extent that they relate to the optical aspect.

## 1 Introduction

As the name photoacoustic tomography suggests, there are two aspects to this emerging imaging modality: an optical part and an acoustic part. This short review paper is focused on the mathematics of the optical part. In particular, it surveys the current thinking regarding two related problems: (1) what is the best way to describe light propagation and its interaction with biological tissue mathematically? (2) Given photoacoustic measurements, what, in principle, can we learn about the optical properties of tissue (or indeed the related, and more clinically relevant, properties, such as blood oxygenation)? Because the ultimate aim is to obtain quantitative estimates of the tissue constituents, this topic is sometimes referred to as quantitative photoacoustic tomography (QPAT).

### 1.1 Photoacoustic Imaging

There are a few closely related but different imaging modalities that come under the heading of photoacoustic imaging. All exploit the photoacoustic effect, which is when a sufficiently short pulse of light is absorbed by an elastic material and subsequently thermalized, the site of the absorption will act as a source of an acoustic pulse.^1^^–^^3^ In all variants, the light pulse is directed into the soft biological tissue under investigation, and the resulting acoustic pulse is measured at the tissue surface. From the measurements of the acoustic pulse, an image of where the light was absorbed can be formed. That is a photoacoustic image. Photoacoustic microscopy differs from photoacoustic tomography in the way the data is collected and the image is formed. In photoacoustic microscopy, either the light beam or the acoustic detector is sharply focused and raster-scanned across the tissue surface.^1^^,^^4^ Because of the localization caused by the focusing, an image can be formed directly from the measured acoustic time series; indeed, it is the tightness of the focusing that determines the resolution of the image. (The fact that the source or detector is commonly raster-scanned is not what makes this microscopy; an array of focused sources or detectors could just as well be used.) In photoacoustic tomography, by contrast, the light is unfocused—indeed, the illumination is arranged such that the whole region-of-interest is flooded with light—and an array of unfocused (or, at least, not tightly focused) detectors is used to record the resulting acoustic time series.^1^^,^^2^ Because the photoacoustic source may be distributed throughout the tissue and because each time series could contain signals from anywhere (as the detectors are unfocused), the connection between the data and source is more complicated than for microscopy, and it is necessary to use an image reconstruction algorithm to form an image. Photoacoustic tomography, not microscopy, is the primary concern of this review, although the tissue optics described will be applicable to all photoacoustic imaging approaches in turbid media.

### 1.2 Scope of the Review

Even with this restriction to photoacoustic tomography, the size of the field has grown rapidly in recent years and it is not possible to review all aspects of it in a short article. We have therefore limited the scope further and discussed the acoustic aspects of photoacoustic tomography only to the extent that they relate to the optical aspects, which is our primary concern. Furthermore, we do not describe experimental photoacoustic tomography, except to note how practical constraints impact the optical inversions (often very significantly) and make few references to specific applications. Finally, we do not provide a comprehensive literature review as that would comprise too long a list, but we hope that the articles we do reference can act as a route into the broader field for the interested reader.

### 1.3 Layout of the Paper

In Sec. 2, the commonly used mathematical models of how light (Sec. 2.1) and sound (Secs. 2.2 and 2.3) propagate in biological tissue are introduced, and in Sec. 3, the corresponding inverse problems are introduced. In particular, Sec. 3.1 briefly overviews how the acoustic inverse problem is usually treated, leading up to the main topic of this review in Sec. 3.2: the optical inverse problem. The review ends with Sec. 4, which highlights some of the ways in which limitations of real-life measurements affects the inverse problems in practice and the trade-offs necessary when using experimental measurements.

## 2 Modeling Photoacoustic Waves

**Fig. 1:**
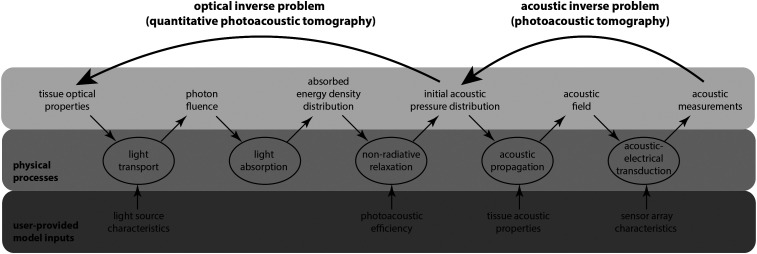
Key stages in QPAT and the related physical phenomena and quantities.

The key physical phenomena and quantities relevant to photoacoustic tomography are shown schematically in Fig. 1. The principal physical phenomenon of relevance to QPAT is light transport; the other physical phenomena are relevant to the inverse problem of obtaining an accurate estimate for the initial acoustic pressure distribution, which acts as the data for the optical part of the problem.

### 2.1 Light Transport

Light propagation in scattering media, such as biological tissue, can be described using transport theory.^5^^,^^6^ In transport theory, light energy conservation within a small volume element of phase space is investigated. Wave phenomena, such as interference, Anderson localization, and enhanced backscattering, are assumed to be negligible and are ignored. Light transport can be modeled using deterministic and stochastic methods. In the deterministic approach, light transport is described with integro-differential equations that can be solved analytically or the solution can be numerically approximated. Generally in QPAT, the radiative transfer equation (RTE) or its approximations are used. The RTE is a “one-speed” approximation of the transport equation, which means it is assumed that the energy (or speed) of photons does not change in collisions (elastic collisions only) and that the refractive index is constant within the medium. For a discussion and studies of photon transport in a medium with spatially varying or piecewise constant refractive index, see Refs. 7–10 and the references therein. In the stochastic approach, the photons’ absorption and scattering interactions with the medium are simulated directly. The most widely applied stochastic methods for simulating light transport in biological tissue are Monte Carlo methods, briefly described in Sec. 2.1.5 (see also reviews Refs. 11 and 12 and the references therein).

#### 2.1.1 Optical absorption and scattering

A photon of light is a rapid fluctuation in the electromagnetic field, which when close to a molecule can induce a dipole moment in the molecule, self-creating a mechanism through which the molecule then interacts with the photon. When the frequency of the fluctuation corresponds to the energy of an allowed transition between two energetic states of the molecule, energy transfer from the light to the molecule—absorption of the photon—is highly likely to occur. Some of the absorbed energy may then be reradiated, e.g., as fluorescence, but the part of interest in photoacoustics is the part converted to heat, typically via collisional relaxation with neighboring molecules, often water molecules. A measure of the likelihood of absorption occurring in a bulk material made up of many of the same molecules is given by the “molar absorption coefficient,” but most biological tissue consists of many different types of molecules, so it is useful to define the overall “absorption coefficient” $\mu_{a}\;(m^{-1},\text{often stated in }{\mathrm{mm}}^{-1})$ of the tissue as a linear sum of the constituent components:^13^^,^^14^

$$\mu_{a}(\lambda )=\overset{K}{\underset{k=1}{\sum }}\alpha_{k}(\lambda )C_{k}(r),$$ (1)

where there are K components, $\alpha_{k}(\lambda )$ and $C_{k}(r)$ are the molar absorption coefficient and concentration of the k’th component, respectively, and λ is the wavelength of the light. The concentrations $C_{k}(r)$, and therefore the absorption coefficient, will in general be spatially varying, where r denotes the spatial position. The absorption coefficient describes the likelihood of a photon being absorbed; specifically, the probability of a photon being absorbed while travelling a short distance ds is $\mu_{a}ds$. Indeed, the absorption coefficient is also the rate in which the intensity of light beam will decay in a purely absorbing (nonscattering) medium, i.e., the intensity will decay as $\exp(-\mu_{a}s)$, where s is the propagation distance.

When the optical frequency is not close to the energy of an allowed transition, an oscillating dipole moment can be induced that reradiates the wave, i.e., scatters the photon in a random direction. In optical transport theory, the probability density function describing the random scattering of incident light from a bulk medium is called the “scattering phase function,” denoted here by Θ(s^,s^′), where the unit vectors $\hat{s}$ and s^′ are the scattered and incident photon directions, respectively. The phase function will depend on the optical wavelength as well as the molecules doing the scattering. The probability that a photon will be scattered while traversing the distance ds is $\mu_{s}ds$, where $\mu_{s}(m^{-1})$ is called the “scattering coefficient.” It is through these three quantities, the absorption and scattering coefficients and the scattering phase function, all wavelengths dependent that the optical properties of tissue are described in transport theory.

#### 2.1.2 Radiative transfer

The RTE can be derived through transport theory^5^ or from Maxwell’s equations.^15^^,^^16^ It describes the distribution of radiance within a domain $\Omega \subset R^{d}$ with boundary ∂Ω as

**Fig. 2:**
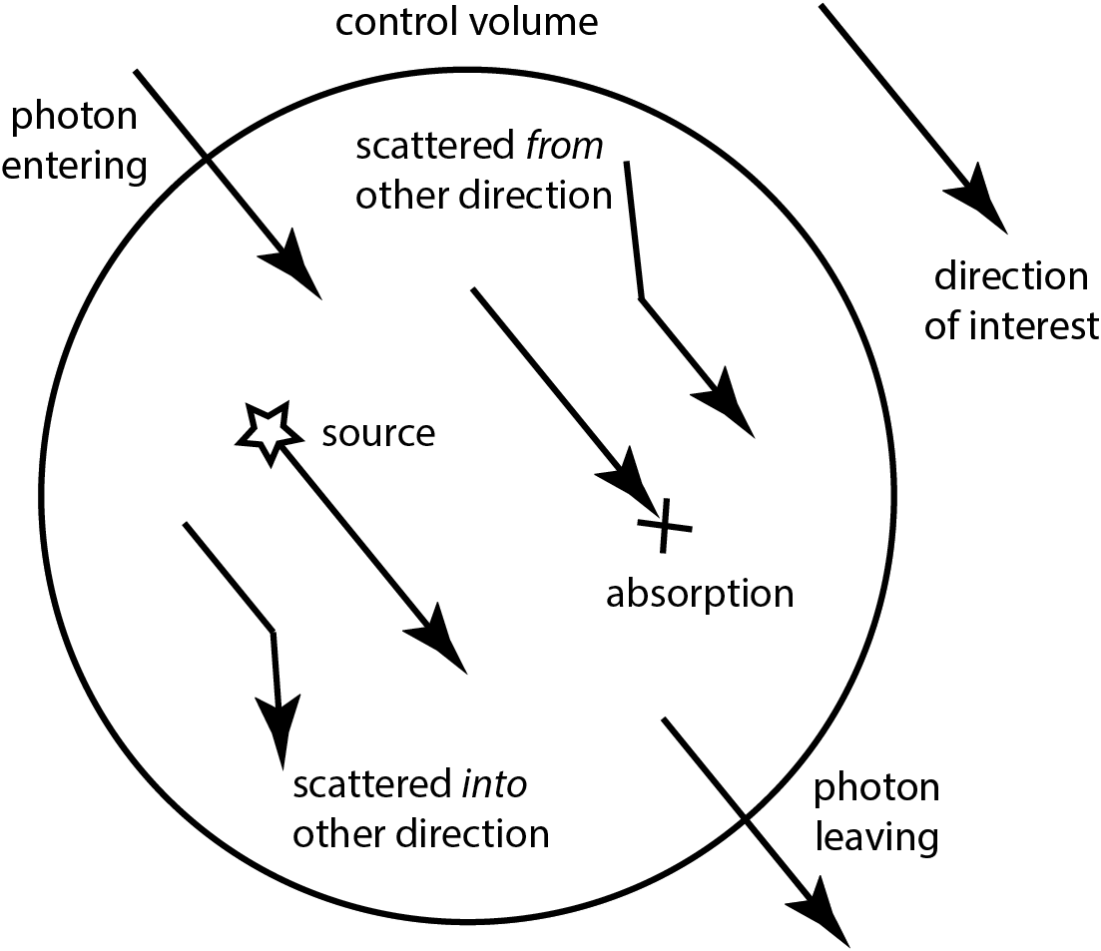
Illustration of the terms in the RTE considered for one direction of propagation $\hat{s}$. The rate of change of the radiance $\phi (\hat{s})$ within the control volume will depend on the net amount of light travelling in direction $\hat{s}$ entering and leaving the volume ($\hat{s}\cdot \nabla \phi$), the photon generated or absorbed within the control volume [q and $\mu_{a}\phi (\hat{s})$, respectively], the photons scattered out of direction $\hat{s}$ into another direction [$\mu_{s}\phi (\hat{s})$], and finally the photons scattered from any other direction into direction $\hat{s}$ (given by the integral term).

{1c∂ϕ(r,s^,t,λ)∂t+s^·∇ϕ(r,s^,t,λ)+(μs(r,λ)+μa(r,λ))ϕ(r,s^,t,λ)=μs(r,λ)∫Sd−1Θ(s^,s^′)ϕ(r,s^′,t,λ)ds^′+q(r,t,λ),  r∈Ωϕ(r,s^,t,λ)={ϕ0(r,s^,t,λ)+Rϕ(r,Ms^,t,λ),r∈rs,  s^·n^<0Rϕ(r,Ms^,t,λ),r∈∂Ω∖rs,  s^·n^<0, (2)

where d is the (spatial) dimension of the domain (d=2 or 3), $\hat{s}\in S^{d-1}$ is a unit vector in the direction of interest, ϕ(r,s^,t,λ)[W/(m2sr)] is the radiance at point r, direction $\hat{s}$, and time instance t, c is the speed of light in the medium, q(r,t,λ) is an internal light source, which can also be placed on the boundary, $\phi_{0}(r,\hat{s},t,\lambda )$ is a boundary light source at the source position $r_{s}\subset \partial \Omega$, and $\hat{n}$ is an outward unit normal (see Fig. 2).

Further, R is the Fresnel reflection coefficient and the mapping M gives the change in light direction due to reflection at the boundary,^8^ thus in the case of matched refractive indices between the medium and surrounding medium, R=0. The radiance can be defined such that the amount of power transfer in the infinitesimal angle $d\hat{s}$ in direction $\hat{s}$ at time t through an infinitesimal area dS is given by

ϕ(r,s^,t,λ)s^·n^dSds^,

where $\hat{n}$ is the unit normal to the surface dS.^5^ The scattering phase function Θ(s^,s^′) describes the probability that a photon with an initial direction s^′ will have a direction $\hat{s}$ after a scattering event. It is often assumed that this depends only on the angle θ between the incoming and outcoming directions, i.e., Θ(s^,s^′)≈Θ(s^·s^′) where s^·s^′=cos θ. In biomedical optical imaging, the most commonly applied phase function is the Henyey–Greenstein phase function,^17^ which is of the form:

ΘHG(s^·s^′)={12π1−g2(1+g2−2  g(s^·s^′)),d=214π1−g2(1+g2−2  g(s^·s^′))3/2,d=3, (3)

where g (dimension less) is the scattering anisotropy parameter that defines the shape of the probability density. It takes values between −1<g<1, such that, if g=0, the scattering probability density is a uniform distribution, g>0 for forward dominated scattering, and g<0 for backward dominated scattering. Experimental measurements made for determining the anisotropy parameter g show that it is typically highly forward scattering in biological tissues.^14^ The Henyey–Greenstein phase function has the useful property that its Legendre expansion is given by powers of the parameter g:

∫Sd−1Pl(τ)ΘHG(τ)ds^′=gl, (4)

and we have an expression:

ΘHG(s^·s^′)={12π+1π∑n=1∞gn cos(n(s^·s^′)),d=2∑l∞∑m=−llglY¯l,m(s^′)Yl,m(s^),d=3, (5)

where d=2 is a Fourier series representation and d=3 is spherical harmonics $Y_{l,m}$ expansion of the phase function.^18^^,^^19^ Although widely applied in biomedical optics, the Henyey–Greenstein phase function may show differences to measured signals in some situations, such as spatially resolved reflectance measurements, where other phase functions could be applied.^20^

As light propagates within the tissue, it is absorbed leading to a localized increase in pressure and generation of a pressure wave. In QPAT, propagation of the acoustic wave occurs on a microsecond time scale, orders of magnitude slower than the optical timescale that includes the optical pulse length as well as the optical propagation, absorption, and the decay of the absorbed optical energy to heat. Therefore, only the total absorbed optical energy density is of interest and not the rate of the absorption. Thus, in QPAT, light propagation can be modeled using a time-independent model for light transport. The time-independent RTE is

{s^·∇ϕ(r,s^,λ)+(μs(r,λ)+μa(r,λ))ϕ(r,s^,λ)=μs(r,λ)∫Sd−1Θ(s^·s^′)ϕ(r,s^′)ds^′+q(r,λ),  r∈Ωϕ(r,s^,λ)={ϕ0(r,s^,λ)+Rϕ(r,Ms^,λ),r∈rs,  s^·n^<0Rϕ(r,Ms^,λ),r∈∂Ω∖rs,  s^·n^<0, (6)

where ϕ(r,s^,λ)=∫−∞∞ϕ(r,s^,t,λ)dt[J/(m2sr)] is the time-independent radiance, and q(r,λ) and $\phi_{0}(r,\hat{s},\lambda )$ are time-independent light sources.

#### 2.1.3 Approximations to the radiative transfer equation

In biomedical optics, popular approximations of the RTE are the diffusion approximation (DA), which is valid when the field is diffuse, and the Beer–Lambert law, which is valid in nonscattering media.

##### Diffusion approximation

The typical approach to derive the DA is to expand the radiance, source term, and phase function into series using the spherical harmonics and truncate the series.^18^ The first-order spherical harmonics approximation is referred to as the $P_{1}$ approximation and the DA can be regarded as a special case of that. In the framework of the DA, the radiance is approximated by

ϕ(r,s^,t,λ)≈1|Sn−1|Φ(r,t,λ)+n|Sn−1|s^·J(r,t,λ), (7)

where $\Phi (r,t,\lambda )\;(W/m^{2})$ and $J(r,t,\lambda )\;(W/m^{2})$ are the photon fluence rate and photon current (flux):

Φ(r,t,λ)=∫Sd−1ϕ(r,s^,t,λ)ds^, (8)

J(r,t,λ)=∫Sd−1s^ϕ(r,s^,t,λ)ds^. (9)

By inserting the approximation (7) and similar approximations written for the source term and phase function into Eq. (2) and following the derivation in Refs. 5 and 21, the DA can be derived

{1c∂Φ(r,t,λ)∂t−∇·κ(r,λ)∇Φ(r,t,λ)+μa(r,λ)Φ(r,t,λ)=q0(r,λ),  r∈ΩΦ(r,t,λ)+12γdκ(r,λ)A∂Φ(r,t,λ)∂n^={Is(r,t,λ)γd,r∈rs0,r∈∂Ω∖rs, (10)

where $\kappa (r,\lambda )={\left(d(\mu_{a}(r,\lambda )+\mu_{s}^{\prime }(r,\lambda ))\right)}^{-1}\;(m^{-1})$ is the diffusion coefficient and where $\mu_{s}^{\prime }(r,\lambda )=(1-g_{1})\mu_{s}(r,\lambda )\;(m^{-1})$ is the reduced scattering coefficient, d is the dimension (d=2 or 3), and $g_{1}$ is the mean of the cosine of the scattering angle that in the case of the Henyey–Greenstein phase function (3) is $g_{1}=g$. It is clear from this that, in highly scattering media, the scattering anisotropy parameter and scattering coefficient merge into a single parameter, the reduced scattering coefficient. Furthermore, $\gamma_{d}$ is a dimension-dependent constant that takes values $\gamma_{2}=1/\pi$ and $\gamma_{3}=1/4$. Parameter $A=(1+R)(1-R{)}^{-1}$ governs reflection on the boundary, with A=1 in the case of no boundary reflection. $q_{0}(r,\lambda )$ is an internal light source that is utilized in biomedical optics in fluorescence diffuse optical tomography (DOT) and in numerical simulations of light transport if an internal point source model is used, and $I_{s}(r,t,\lambda )$ is a diffuse boundary source. It should be noted that, in biomedical optics, the boundary condition of the DA is sometimes numerically implemented by applying a Diriclet boundary condition on a “virtual boundary” outside the domain (so-called extrapolated boundary condition).^22^

As in the case of the RTE, the time-independent DA can be derived. It takes the form:

{−∇·κ(r,λ)∇Φ(r,λ)+μa(r,λ)Φ(r,λ)=q0(r,λ),  r∈ΩΦ(r,λ)+12γdκ(r,λ)A∂Φ(r,λ)∂n^={Is(r,λ)γd,r∈rs0,r∈∂Ω∖rs, (11)

where $\Phi (r,\lambda )=\int_{-\infty }^{\infty }\Phi (r,t,\lambda )dt\;(J/m^{2})$ is the (time-independent) fluence and $q_{0}(r,\lambda )$ and $I_{s}(r,\lambda )$ are time-independent light sources.

The DA is valid when the radiance is almost a uniform distribution [see approximation of radiance Eq. (7)]. In other words, when the radiance is almost independent of direction; the light reaching any point is coming from all directions. In practice, this is achieved in a scattering-dominated medium further than a few scattering lengths from light sources.^5^ In many QPAT experiments, however, the imaging depth can be small compared to the average scattering length, and thus the DA is not always a valid approximation.

##### Beer–Lambert law

If the medium is nonscattering (purely absorbing) and if the radiance is collimated (propagating in one direction), the RTE becomes

(s^·∇)ϕ(r,s^,λ)=−μa(r,λ)ϕ(r,s^,λ), (12)

from which it is clear that the decrease in intensity through a purely absorbing medium in direction z can be written as

$$d\Phi (z,\lambda )=-\mu_{a}(z,\lambda )\Phi (z,\lambda )dz.$$ (13)

If the source is a monodirectional flux $\Phi_{0}(r,\lambda )$ incident in the positive z-direction, Eq. (13) has the solution:

$$\Phi (r,\lambda )=\Phi_{0}(r,\lambda )\exp(-\int_{z_{0}}^{z}\mu_{a}(z^{\prime },\lambda )dz^{\prime }),$$ (14)

which is known as the Beer–Lambert law.

A second scenario, in which exponential decay of the light flux occurs, is when a plane wave is incident on a homogeneous scattering medium for which the DA holds. In this case, Eq. (11) becomes

$$\frac{d^{2}\Phi (z,\lambda )}{dz^{2}}=(\frac{\mu_{a}}{\kappa })\Phi (z,\lambda ),$$ (15)

which has solution

$$\Phi (z,\lambda )=\Phi_{0}(z,\lambda )\exp(-\mu_{\mathrm{eff}}z),\;\mu_{\mathrm{eff}}=\sqrt{\mu_{a}/\kappa },$$ (16)

where z is the coordinate perpendicular to the propagation front of the plane wave. This approximation and similar approximations exploiting the parameter $\mu_{\mathrm{eff}}$ have been used in a number of QPAT studies.^23^^–^^28^

##### Other approximations

In addition to DA, other orders of the spherical harmonics expansion $P_{N}$ and approximations made for them, such as simplified spherical harmonics,^29^ have been utilized in biomedical optics. Furthermore, hybrid models coupling nonscattering and/or low-scattering medium with highly scattering medium have been developed.^30^^–^^34^ In QPAT, utilizing simplified spherical harmonics approximation has been utilized.^35^^,^^36^ Numerical approximation of the RTE with Henyey–Greenstein phase function can be challenging if scattering is highly forward dominated. Therefore, approximations to the RTE that takes into account forward-peaked scattering analytically have been proposed. These include the delta-Eddington approximation, the Fokker–Planck approximation, the Fokker–Planck–Eddington approximation and the generalized Fokker–Planck–Eddington approximation.^32^^,^^37^^,^^38^

#### 2.1.4 Numerical approximations

The analytical solutions of the RTE and the DA are typically limited to specific geometries, and therefore their utilization in biomedical optical imaging has been limited. Therefore, the typical approach has been to numerically approximate their solutions. For the numerical approximation of the DA, the most widely applied approach has been to use the finite-element method,^22^^,^^39^ with free software also available.^40^^,^^41^ The numerical approaches for the RTE include finite difference,^42^^,^^43^ finite element,^33^^,^^44^^,^^45^ and finite volume^46^ methods for spatial discretization. The numerical stability of these has been advanced using special basis functions, such as the streamline diffusion modification.^33^^,^^47^ In addition, numerical approximation using the discontinuous Galerkin method has been implemented.^48^ For angular discretization, discrete ordinates,^42^^,^^43^^,^^46^ finite elements,^33^ and spherical harmonics^44^^,^^45^ have been used. Recently, the pseudospectral method for the numerical approximation of the RTE was proposed.^49^ Recently, many computational challenges related to the numerical approximation of the RTE have been overcome through the development of computing resources and numerical methods such as parallel computing and preconditioning.^50^^,^^51^

#### 2.1.5 Monte Carlo method for light transport

The Monte Carlo method for light transport can be used to simulate the propagation of photons in a scattering medium in which they undergo random absorption and scattering events.^52^^,^^53^ The methodology has been utilized in a variety of applications in biomedical optics, and different implementations of the Monte Carlo method exist^11^^,^^12^^,^^54^^,^^55^ together with various free and/or open access software available.^53^^–^^60^ In addition to depositing the energy (or photon fluence), it is also possible to record the direction of an absorbed photon (packet). This is sometimes called radiance Monte Carlo.^61^^–^^63^

Monte Carlo simulation in a biological tissue obeys the following principles.^11^^,^^52^^,^^53^ The scattering length follows an exponential probability distribution function:

$$f(l)=\mu_{s}(l)\exp(-\int_{0}^{l}\mu_{s}(l^{\prime })dl^{\prime }).$$ (17)

Then in the case of a scattering event, the scattering angle follows a probability distribution for scattering direction that in many studies is the Henyey–Greenstein phase function (3). For absorption, a general approach in biomedical optics has been to use a so-called photon packet method.^52^ In this approach, a photon packet with an initial weight $w_{0}$ is simulated. As the photon packet propagates, its weight along trajectory is reduced due to absorption, and the photon weight can be described as

$$w(s)=w_{0}\;\exp(-\int_{0}^{s}\mu_{a}(s^{\prime })ds^{\prime }).$$ (18)

where $\mu_{a}(s^{\prime })$ is the absorption coefficient along the trajectory of the photon packet. This is continued until the photon packet exits the computation domain, or its weight becomes negligible. Sampling scattering lengths from Eq. (17) with a weight factor assigned to those paths according to Eq. (18) is a form of importance sampling.^64^^,^^65^

In QPAT, the absorbed optical energy density $H_{j}$ in a discretization element j of the computation domain can be computed as

$$H_{j}=-\frac{1}{A_{j}}\int_{0}^{s_{t}}\chi_{j}(s)\frac{dw}{ds}(s)ds,$$ (19)

where $A_{j}$ is the area or volume of the discretization element j, the integral is understood as being carried from source position where the photon packet was created (s=0) until where it is terminated ($s=s_{t}$), $\chi_{j}$ is the characteristic function of j’th element, and $-\frac{dw}{ds}(s)$ is the energy absorbed by the medium during the photon packet propagation.^64^^,^^66^

### 2.2 Linking Light and Sound: the Photoacoustic Efficiency

Ultimately, the photons that have been multiply scattered around in the tissue either leave the tissue or are absorbed by chromophores (light-absorbing molecules). The resulting absorbed optical energy density $H(r,\lambda )\;(J/m^{3})$ can be written as

$$H(r,\lambda )=\mu_{a}(r,\lambda )\Phi (r,\lambda ),$$ (20)

where Φ(r,λ) is the photon fluence as described by Eqs. (6), (8), and (11). Assuming there is no reradiation such as fluorescence (often a good assumption for tissue), all the energy thermalizes into heat. For efficient acoustic generation, the optical pulse must be short enough that the tissue does not have time to deform significantly (an isochoric condition). This results in a localized pressure increase, often termed the “initial acoustic pressure distribution” $p_{0}(r)\;(\mathrm{Pa})$ as it is this that leads to the acoustic pressure pulse that travels to the tissue surface. The absorbed optical energy density is linked to the initial acoustic pressure distribution through the photoacoustic efficiency, which can be identified with the Grüneisen parameter G(r)(dimensionless) for a pure optically absorbing fluid:

$$p_{0}(r)=G(r)H(r,\lambda )=\frac{\beta v^{2}}{C_{p}}H(r),$$ (21)

where β ($K^{-1}$) is the volume thermal expansivity of the fluid, v (m/s) is the speed of sound, and $C_{p}$ (J/kg/K) is the specific heat capacity at constant pressure.^1^^–^^3^ It is worth noting that the Grüneisen parameter can depend on the wavelength,^67^ although it is usually treated as constant and can also depend of the concentration of the absorber^68^^–^^70^ and the temperature.^71^^,^^72^

### 2.3 Acoustic Initial Value Problem

This paper, as mentioned in Sec. 1, is primarily concerned with optical aspects of photoacoustic tomography, and so this section on the acoustic propagation will be a brief summary. In photoacoustic tomography, the acoustic amplitudes are sufficiently low that the propagation of the photoacoustic wave can be modeled using equations of linear acoustics. For soft biological tissue, it is generally assumed that the medium is acoustically isotropic and quiescent. Shear waves can be neglected because they are not strongly generated by the photoacoustic effect; furthermore, the speed of shear wave propagation is orders of magnitude lower than the compressional wave speed. In a lossless medium, the linear acoustic wave equation can be written as

$$\frac{1}{v(r{)}^{2}}\frac{\partial^{2}p(r,t)}{\partial t^{2}}+\frac{1}{\rho_{0}(r)}\nabla \rho_{0}(r)\cdot \nabla p(r,t)-\nabla^{2}p(r,t)=0,$$ (22)

where p(r,t)(Pa) is the acoustic pressure, v(r) is the speed of sound, and $\rho_{0}(r)$ is the ambient mass density. (In the case of constant ambient density and constant speed of sound, it reduces to the canonical wave equation.) Combining the wave equation with the initial conditions relevant to photoacoustic wave generation:

$$p(r,t=0)=p_{0}(r),\;\frac{\partial p(r,t=0)}{\partial t}=0,$$ (23)

gives an acoustic initial value problem. A spatially varying sound speed is often taken into account in photoacoustic models,^73^^–^^77^ although few consider spatially varying mass density. The effect of acoustic attenuation, not modeled by the above equation, has also been considered in Refs. 78–81. Furthermore, modeling wave propagation in elastic media, such as bones, has been considered in Refs. 82–85. Several numerical models are freely available for simulating photoacoustic waves and for use in solving the inverse problem.^86^^–^^92^

As real ultrasound detectors are, sadly, never point-like and instantaneously responsive pressure sensors, the measured data will not be the acoustic pressure but a filtered version of it (filtered in both temporal and spatial frequency space):

$$p_{d}(r_{d},t)=M(p(r,t){|}_{r=r_{d}}),$$ (24)

where $r_{d}$ are the nominal positions of the detectors. The linear filter M can be usefully approximated in many cases as the separable product of a temporal and a spatial filter, often referred to as the frequency response and the directionality of the detector, respectively. Accounting for these filtering effects can be important when tackling the inverse problem (see Sec. 4). There is a large literature on modeling ultrasound sensor responses.^93^^,^^94^

## 3 Photoacoustic Inverse Problems

In the inverse problem of QPAT, the aim is to estimate the spatially varying concentrations of light absorbing molecules (or related quantities) when the measured photoacoustic time series and the parameters of input light are given. As mentioned earlier, the difference in time scales of light absorption and ultrasound propagation allows the optical and acoustic parts of the problem to be decoupled. Thus the related inverse problems can be treated separately. The two inverse problems in QPAT then are: (1) acoustic inverse problem, that is, estimate the initial acoustic pressure distribution $p_{0}(r)$ from measured photoacoustic time-series and (2) optical inverse problem, that is, estimate the distributions of the optical parameters from the absorbed optical energy density (or the initial pressure).

When the two inverse problems are treated separately, and the output of the acoustic inversion becomes the data for the optical inversion, the quality of the acoustic inversion is clearly critical to the success of the optical inversion.^95^

### 3.1 Acoustic Inverse Problem

Before an experimental photoacoustic system is constructed, a choice must be made regarding the trade-off between the speed with which the data will be acquired and the potential accuracy of the image. (There are, of course, other considerations that affect this decision, such as cost, availability of equipment, and clinical restraints.) Without sufficient data it will never be possible to reconstruct an accurate image. However, supposing that sufficient data is available, a second and related choice must be made when trying to estimate the initial acoustic pressure distribution $p_{0}(r)$ from the measured data $p_{\mathrm{meas}}(r_{d},t)$, which is regarding the trade-off of accuracy of the image and the speed with which the image is computed. There are multiple ways in which the many approaches to photoacoustic image reconstruction can be categorized, and none are perfect, but for this review, we consider three broad categories: (1) methods that are based on an analytical formula, (2) methods that rely on a numerical acoustic model, and (3) data-driven methods.

Under the first category fall all filtered backprojection-type methods,^96^^–^^98^ which includes beamforming methods, and which relate to the inverse spherical mean Radon transform. These can be coded up to be very fast, as they require just (pre- and sometimes post-) filtering and a backprojection step, which maps the filtered data directly into an image. It also includes methods that are based on (truncated) series formulas, which can also be made fast in some cases.^99^^,^^100^ However, these methods are restricted to certain measurement-surface geometries and they typically assume the speed of sound is a constant. Also incorporating a complex directional response for the detectors is nontrivial.

These problems can be overcome by methods that use numerical approximations of acoustic models. Under this category falls time-reversal methods,^81^^,^^101^^–^^104^ regularized least squares,^83^^,^^105^^–^^113^ maximum entropy,^114^ and Bayesian approaches.^115^^–^^118^ The models powering these approaches can be very general and can in principle incorporate the modeling of any aspect of the data acquisition process, although these may have knock-on implications for solving the inverse problem so some parsimony is recommended. Furthermore, with the latter two approaches it is possible to incorporate some types of prior knowledge of the solution to guide the inversion. Common among these are smoothness and total-variation constraints. Moreover, the Bayesian approach can even take into account the uncertainties of parameters, models and geometries.^115^^,^^119^^,^^120^ When the number of detectors and image voxels is small, it can be possible to code the model in a matrix form which can be executed rapidly. However, to obtain accurate images for large imaging volumes with fully sampled measurement surfaces, these methods can be slow to compute as the photoacoustic wavefield within the entire domain needs to be computed at multiple iterations.

Data-driven approaches,^121^^–^^123^ the third category, can overcome both these limitations by learning, in advance, what kind of image is expected, leading to a fast reconstruction at run-time. (The word “data” in “data-driven” is not referring to the experimental data $p_{\mathrm{meas}}(r_{d},t)$ but to a training set of images or raw data, assumed to be drawn from the same distribution as the required image or data, which are available prior to the reconstruction.) In a so-called end-to-end framework, a neural network is trained to estimate the initial pressure distribution based on a set of photoacoustic data.^124^^,^^125^ However, because this mapping is nonlocal (the acoustic waves from anywhere can reach the detectors), this will require a large interconnected network, which will require a large training set. A better approach is to incorporate learned components either before or after a backprojection step. In the preprocessing approaches, a photoacoustic dataset is first processed using a neural network to improve the signal quality and then a photoacoustic image is reconstructed using a backprojection method.^126^^,^^127^ In the postprocessing approaches, a (rough) photoacoustic image is first reconstructed and then a neural network is used to correct artifacts or noise in the reconstructed image.^127^^–^^129^ The trade-off to these advantages, however, is the risk that the learned components hallucinate image features, which are not consistent with the measured data. Various ways to mitigate these disadvantages, such as combining aspects of the above methods, are under investigation.^130^^–^^137^

The short treatment of the acoustic inversion here should not be taken to suggest that it is unimportant with regard to the optical inversion. Accurate solutions to the acoustic inversion are critical to obtaining accurate solutions to the optical inversion, as they provide the data for it. With this in mind, some practical considerations that should be considered when dealing with experimental data are described below in Sec. 4. For more information on image reconstruction methods in PAT see reviews^138^^–^^140^ and the references therein.

### 3.2 Optical Inverse Problem

The optical inverse problem of QPAT is to estimate the spatially varying absorption coefficient, or the spatially varying chromophore concentrations, or related quantities, such as haemoglobin oxygenation state from the estimated initial pressure distribution with a given input light illumination. If the Grüneisen parameter is assumed to be known, the key task is to determine the photon fluence, Φ(r,λ) [see Eq. (20)]. This, however, is complicated by the fact that fluence itself is dependent on the unknown optical properties. This inversion, unlike the acoustic inversion, is therefore nonlinear.

**Fig. 3:**
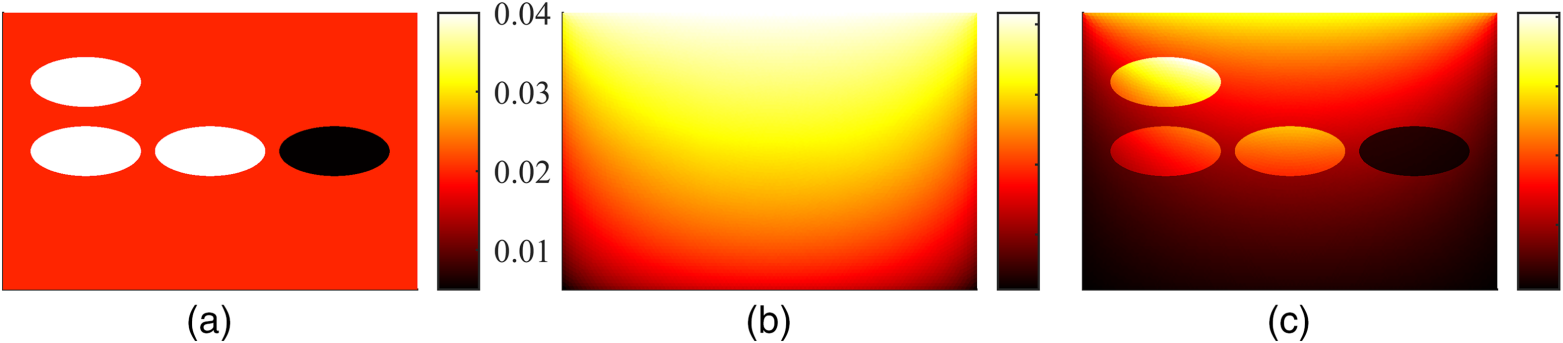
Simulated photoacoustic data with a spatially varying absorption $\mu_{a}\in [0.005,0.04]\;{\mathrm{mm}}^{-1}$ and a constant scattering $\mu_{s}=4\;{\mathrm{mm}}^{-1}$. (a) Absorption coefficient $\mu_{a}({\mathrm{mm}}^{-1})$, (b) logarithm of photon fluence log Φ (arbitrary units), and (c) absorbed optical energy density H (arbitrary units). Image courtesy of N. Hänninen.

**Fig. 4:**
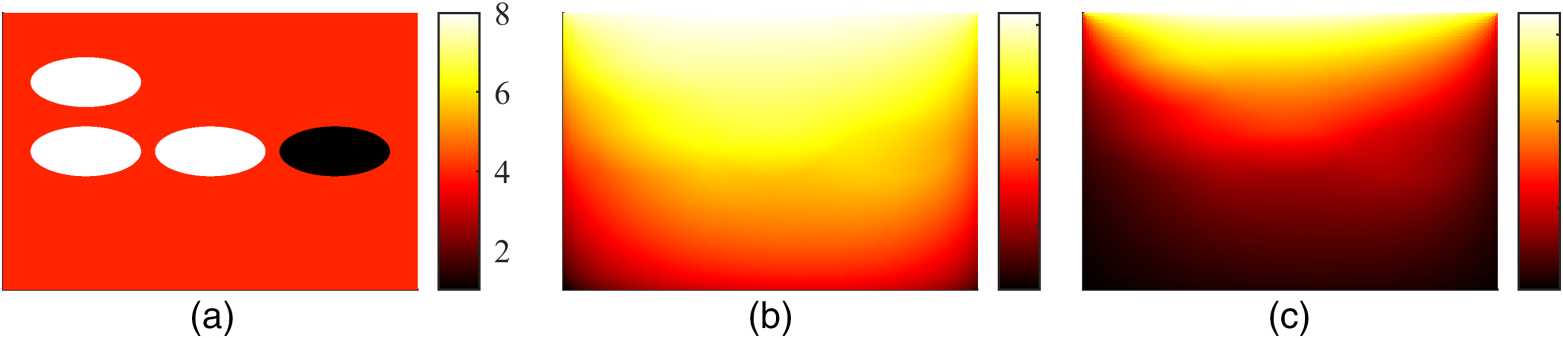
Simulated photoacoustic data with a spatially varying scattering $\mu_{s}\in [1,8]\;{\mathrm{mm}}^{-1}$ and a constant absorption $\mu_{a}=0.02\;{\mathrm{mm}}^{-1}$. (a) Scattering coefficient $\mu_{s}({\mathrm{mm}}^{-1})$, (b) logarithm of photon fluence log Φ (arbitrary units), and (c) absorbed optical energy density H (arbitrary units). Image courtesy of N. Hänninen.

An example of Monte Carlo simulated data in a 2D slab of size 15  mm×10  mm with a spatially varying absorption coefficient $\mu_{a}\in [0.005,0.04]\;{\mathrm{mm}}^{-1}$ and a constant scattering $\mu_{s}=4\;{\mathrm{mm}}^{-1}$, and an anisotropy parameter g=0.9 is shown in Fig. 3. The domain was illuminated with a planar illumination from the top of the domain with a spatially constant and angularly cosine radiance distribution. This figure shows the dependence of fluence Φ on absorption distribution, and the effect of both absorption and fluence on absorbed optical energy density H within the domain, demonstrating the nonlinear nature of the inverse problem of estimating the optical parameters. A similar simulation with a spatially varying scattering $\mu_{s}\in [1,8]\;{\mathrm{mm}}^{-1}$ and a constant absorption $\mu_{a}=0.02\;{\mathrm{mm}}^{-1}$ is shown in Fig. 4. As it can be seen, also scattering affects both the fluence distribution Φ and absorbed optical energy density H. This change in the absorbed optical energy density distribution is, however, less clear than the change due to the absorption, which indicates that reconstruction of scattering is more ill-posed than reconstruction of absorption.

Different methods have been used to approach the optical inverse problem of QPAT. Here we give an overview of those. Many of the methods described here can be described as “iterative methods” since iterative optimization algorithms are utilized in their solution. In addition to these, so-called direct approaches for estimation of optical parameters have also been developed.^141^^–^^143^ For other reviews on the optical inverse problem of QPAT, see Refs. 2, 144, and 145.

There are two possible approaches to the optical inverse problem. Either the chromophore concentrations can be estimated directly from absorbed optical energy density data obtained using multiple wavelengths of light,^69^^,^^142^^,^^143^^,^^146^^–^^149^ or they can be estimated in two stages, first by recovering the absorption coefficients at different wavelengths and then calculating the concentrations with a subsequent, linear, spectroscopic inversion.^2^^,^^142^^,^^146^^,^^147^ In both cases, the molar absorption spectra of the contributing chromophores need to be known a priori. Spectroscopic inversions are well understood mathematically, and they are used in many areas of optics, so we focus mostly in this section on the inversion for the optical parameters at a single wavelength. However, given its practical importance, a brief reminder of the spectroscopic inversion is given below.

#### 3.2.1 Spectroscopic inversion

A very important source of contrast in medical applications of PAT is hemoglobin. First, because it is easy to measure as it absorbs strongly in the near-infrared where the optical scattering is low and there are not too many other competing absorbers. Second, because—at least in principle—it gives a route to forming spatially resolved images of blood oxygen saturation, which is a key indicator of tissue function and pathology. The oxygen saturation is the ratio:

$${\mathrm{sO}}_{2}=C_{{\mathrm{HbO}}_{2}}/(C_{{\mathrm{HbO}}_{2}}+C_{\mathrm{Hb}}),$$ (25)

where $C_{{\mathrm{HbO}}_{2}}$ and $C_{\mathrm{Hb}}$ are the concentrations of the chromophores oxyhemoglobin and deoxyhemoglobin. The link between these concentrations and the initial acoustic pressure distribution $p_{0}(r)$ is given by Eqs. (1), (20), and (21). For the inversion of Eq. (1) to be well-conditioned, it is important to choose wavelengths in which the molar absorption coefficients form linearly independent vectors. It is usually assumed that the Grüneisen parameter is independent of wavelength in this scenario, and therefore cancels out when forming the ratio [Eq. (25)]. Concerningly, it is often assumed in experimental studies that the fluence too is independent of wavelength that the photoacoustic amplitudes are directly proportional to the absorption coefficients, but this cannot be the case. Because of the diffusive nature of the light propagation, the fluence will only be independent of wavelength if the optical properties of the tissue also remain constant with wavelength, in which case a spectroscopic inversion will not be possible. Solving the inverse problems, as described below, is therefore, critical to obtaining accurate estimates of this clinically important parameter.

#### 3.2.2 Uniqueness

Considering the optical inversion at a single wavelength, the simultaneous estimation of more than one optical parameter, e.g., absorption and scattering coefficients, is nonunique if only one light illumination pattern is used.^146^^,^^150^ There are broadly two ways to overcome such ill-posedness: obtain more data to use in the inversion or make assumptions (perhaps based on adjunct data).

The simplest approach has been to assume that the scattering is known and to estimate only the absorption.^151^^–^^156^ Although widely used, the validity of this assumption is questionable in many cases of practical interest, e.g., when the scattering coefficient varies with tissue type or is not known accurately *a priori*. This approach can be improved by modeling the errors caused by the fixed scattering assumption by using a Bayesian approximation error modeling.^157^ When using multiple wavelength data, assumptions can be made about the wavelength dependence^146^^,^^151^ to overcome the nonuniqueness. Several authors have reduced the parameter space by assuming that the optical properties are piecewise constant.^76^^,^^158^^–^^161^

In Ref. 141, it was shown that the nonuniqueness can be overcome using multiple optical illuminations to obtain more data. One way to achieve this would be by illuminating the target from different directions^141^^,^^143^^,^^150^^,^^162^^–^^168^ or using light patterns.^169^ This approach has not yet been exploited experimentally, perhaps because experimentalists are concerned to obtain the maximum signal possible given the weakness of the photoacoustic effect, and so try to flood the tissue with light from as many directions as possible. Another approach that uses more data, but in this case also requires more hardware, is to combine QPAT with another modality to overcome the nonuniqueness, e.g., using DOT^170^^–^^173^ or acousto-optic tomography^174^ to estimate the fluence or using an additional modality to provide structural information^76^^,^^161^ for the light model.

As mentioned earlier, here we address estimation of optical parameters when absorbed optical energy density is given as data, which has been the approach in most research on the optical inverse problem of QPAT. This basically means that the acoustic inverse problem has been solved and the Grüneisen parameter is known. The Grüneisen parameter, however, is typically not known and to make the problem even more complicated, it is a spatially varying parameter that depends on chromophore concentration and temperature, and possibly even on wavelength as discussed in Sec. 2.2. One possibility to approach the problem of unknown Grüneisen parameter would be estimating it simultaneously with the optical parameters. In Ref. 142, it was shown that absorption coefficient, diffusion coefficient and the Grüneisen parameter can be estimated if data at multiple wavelengths are used and a prior information of the unknown parameters take specific forms. For example, the dependence of the coefficient on the spatial variable and on the wavelength variable needs to be separated for stable reconstruction. The recovery of the Grüneisen parameter simultaneously with the optical parameters in the case of multiwavelength optical inverse problem of QPAT has been addressed in Refs. 147 and 172. Further, estimation of absorption and scattering together with mapping of the temperature distribution has been studied in Ref. 175.

#### 3.2.3 Known scattering

When the scattering is known, a simple iteration is available for estimating the absorption coefficient. Rearranging Eq. (20) gives the fixed-point iteration:

$$\mu_{a}^{\left(n+1\right)}(r,\lambda )=H(r,\lambda )/\Phi (r,\lambda ;\mu_{a}(r,\lambda {)}^{\left(n\right)}).$$ (26)

Any suitable model can be used to calculate the fluence from the latest estimate of the absorption coefficient.^153^^,^^156^ While straightforward to implement, this iteration can become sensitive to noise where the fluence is low. An improved method that finds $\mu_{a}$ by minimizing $\left\|\mu_{a}(r,\lambda )\Phi (r,\lambda )-H(r,\lambda )\right\|$ is proposed in Ref. 176.

#### 3.2.4 Regularized least squares methods

Generally in parameter estimation problems, the unknown parameters of interest are determined by minimizing the least squares difference between the measured data and predictions of the forward model. Let us denote unknown optical parameters in a point r by x(r). Further, denote the measurements by a finite dimensional vector $y\in R^{m}$, where m is the number of data points and the forward operator that maps the unknown parameters to data by F(x(r)). Typically, in practical implementations, the parameters and the forward mapping are represented in discrete vector spaces $x(r)\mapsto x\in R^{n}$, $F\mapsto f:R^{n}\mapsto R^{m}$, where n is the number of unknown parameters.

In the optical inverse problem, it has generally been assumed that the data y are the absorbed optical energy density that has been obtained as the solution of the acoustic inverse problem. Most of the methods developed for regularized least squares and the Bayesian approach (described in the next section) in QPAT have used numerical approximation of the DA as the forward operator. In addition, the RTE has also been utilized.^143^^,^^156^^,^^166^^,^^167^^,^^169^^,^^177^

Estimation of the unknown optical parameters can be written as a minimization problem:

$$\arg\;\min\;\frac{1}{2}{\left\|y-f(x)\right\|}^{2}.$$ (27)

In QPAT, estimation of absorption and scattering is generally ill-posed, and they cannot be solved using Eq. (27). The ill-posed nature of the problem can be alleviated by introducing a regularizing penalty term.^178^ In that case, the minimization problem becomes

$$\arg\;\min\;\frac{1}{2}{\left\|y-f(x)\right\|}^{2}+B(x),$$ (28)

where B(x) is the regularizing penalty functional. The classical regularization methodology is to use Tikhonov regularization. In a widely applied generalized Tikhonov regularization, the penalty functional has the form:

$$B(x)=\alpha {\left\|D(x-x_{*})\right\|}_{2}^{2},$$ (29)

where α is a regularization parameter, D is a regularization matrix, and $x_{*}$ is the prior estimate of x. Depending of the choice of the regularization matrix, Tikhonov regularization can enhance solutions with smaller norms by an identity matrix or smooth solutions through usage of difference matrix. In QPAT, Tikhonov regularization has been utilized in Refs. 141–143 and 166.

Since many biological structures are better described as being piecewise constant, other regularizing norms (instead the second norm used in Tikhonov regularization) have been developed. An approach for supporting piecewise regular structures with sharp boundaries is to use total variation regularization that in a continuous formulation can be written as

$$B(x(r))=\beta {\left\|\nabla x(r)\right\|}_{1},$$ (30)

that has been utilized in QPAT in Refs. 141, 164, 167, and 179. Furthermore, sparsity supporting $\ell_{1}$ regularization:

$$B(x(r))=\gamma {\left\|Wx(r)\right\|}_{1},$$ (31)

where β is a regularization parameter, and W is a sparse regularization operator has been utilized.^180^ In addition, penalty acting as a physical limitation of the estimated parameters positivity constraint:

$$B(x{)}_{+}=\Pi_{k=1}^{n}\theta (x_{k}),\;\theta (t)=\{\begin{array}{cc} 1, & t\geq 0\\ 0, & \text{otherwise} \end{array}$$ (32)

can be included into the minimization problem. A positivity constraint can be implemented in the minimization algorithm using projection or penalty methods.^181^

Alternatively to approaching the problem as a nonlinear optimization problem, in Refs. 182 and 183, it was proposed to extract absorption and photon fluence using a sparse signal representation. Then the resulting problem was approached as a linear problem of recovering the absorption and photon fluence without utilizing the forward model for light transport.

#### 3.2.5 Bayesian approach

In the framework of Bayesian inverse problems, the inverse problem is approached using statistical inference.^115^ All parameters are modeled as random variables, and information about them is expressed by probability distributions. The observation model with an additive noise model is of the form:

y=f(x)+e, (33)

where $e\in R^{m}$ denotes the noise. In the inverse problem, information about the parameters of primary interest is obtained based on the measurements, the model, and the prior information about the parameters. The solution of the inverse problem is the posterior probability distribution π(x|y) that according to Bayes’ theorem can be presented as a conditional probability density function of the form:

π(x|y)∝π(y|x)π(x), (34)

where π(y|x) is the likelihood density and π(x) is the prior density. Assuming the noise e and the unknown x mutually independent, observation model Eq. (33) leads to a likelihood density:

$$\pi (y|x)=\pi_{e}(y-f(x)),$$ (35)

where $\pi_{e}(\cdot )$ is the probability density of the noise. Let us further model the noise e and unknown optical parameters x as Gaussian distributed, i.e., $e∼N(\eta_{e},\Gamma_{e})$ and $x∼N(\eta_{x},\Gamma_{x})$, where $\eta_{e}$ and $\Gamma_{e}$ are the mean and covariance of the noise, and $\eta_{x}$ and $\Gamma_{x}$ are the mean and covariance of the prior. In this case, the posterior probability density can be written as

$$\pi (x|y)\propto \exp\{-\frac{1}{2}{\left\|L_{e}(y-f(x)-\eta_{e})\right\|}^{2}-\frac{1}{2}{\left\|L_{x}(x-\eta_{x})\right\|}^{2}\},$$ (36)

where $L_{e}^{T}L_{e}=\Gamma_{e}^{-1}$ and $L_{x}^{T}L_{x}=\Gamma_{x}^{-1}$ are the square roots, such as the Cholesky decompositions of the inverse covariance matrices of the noise and prior, respectively.

In principle, the distributions of the unknown parameters can be estimated using Markov chain Monte Carlo methods.^184^ However, these methods can be prohibitively computationally too expensive in large dimensional tomographic inverse problems. Therefore, point estimates are computed to approximate the posterior distribution. An often considered point estimate in tomographic imaging is the *maximum a posteriori* (MAP) estimate

$$x_{\mathrm{MAP}}=\arg\;\min_{x}\{\frac{1}{2}{\left\|L_{e}(y-f(x)-\eta_{e})\right\|}^{2}+\frac{1}{2}{\left\|L_{x}(x-\eta_{x})\right\|}^{2}\}.$$ (37)

If the forward operator is linear and in the case of Gaussian noise and prior, the MAP estimate is also the mean of the posterior. However, as mentioned earlier, optical inverse problem of QPAT is nonlinear.

If the entire posterior distribution could be solved, it can be used to evaluate the reliability of the estimated parameters through examining the standard deviation. In the case of a nonlinear inverse problems, where (only) the MAP estimate has been solved, the standard deviation can be approximated through Laplace’s approximation, leading to a Gaussian approximation for the posterior distribution in the location of the MAP estimate

x|y∼N(x^,Γ^),

where $\hat{x}$ is the MAP estimate and

Γ^=(J(x^)TΓe−1J(x^)+Γx−1)−1 (38)

is the covariance, where $J(\hat{x})$ is the Jacobian matrix of f(x).

The Bayesian approach provides a natural methodology for taking into account the uncertainties in parameters, data, and models; thus Bayesian approximation error modeling^115^ has been utilized in modeling of uncertainties in various applications. The Bayesian approach was formulated for the optical inverse problem of QPAT in Ref. 116, where also modeling of noise and errors due to the acoustic solver were studied. The approach has been further utilized in marginalization of scattering in Ref. 157 and it has been extended for spectral QPAT in Ref. 147.

#### 3.2.6 Learned/data-driven methods

As for the acoustic inverse problem described above, and biomedical imaging in general, data-driven learning-based approached have gained interest also for the optical inverse problem of QPAT (see Refs. 121, 122, and 185–187 and the references therein). Many of the studies are numerical simulation studies using U-Nets or variants to map directly from synthetic maps of absorbed energy density to optical coefficients of interest, including blood oxygenation.^188^^–^^196^ A hybrid approach is to use a learned segmentation algorithm to determine the tissue model and then model the fluence as above.^159^ Although these numerical studies seem promising, the domain gap between the simulated datasets used for training and real measured data casts doubt on how well these methods will translate to experimental settings. Some attempts have been made to address this recently. One approach tackles the issue by aiming to improve the quality of synthetic datasets through the use of detailed anatomical digital phantoms.^197^^,^^198^ Another promising approach is to make measured datasets on physical phantoms and use those for training.^191^ A third attempt tries to close the domain gap using GANs to move the simulated data distribution closer to the real data distribution.^199^^,^^200^

#### 3.2.7 Utilizing Monte Carlo method in the inverse problem of QPAT

Recently, utilization of Monte Carlo method for light transport in the optical inverse problem of QPAT has raised interest. The methodology has been utilized in estimating absorption while assuming scattering as known.^201^^,^^202^ Utilizing Monte Carlo in the minimization approaches, such as regularized least squares or MAP estimation, for estimating both absorption and scattering in QPAT requires evaluation of a minimization direction, such as the steepest descent or Gauss–Newton direction. The gradients for a steepest descent algorithm can be obtained using a so-called adjoint Monte Carlo.^62^ The approach has been utilized in estimating absorption^61^^,^^62^^,^^203^ and it has also been evaluated with experimental data.^204^ Alternatively, Jacobian matrices can be constructed by computing the derivative for the absorption coefficient directly from Eq. (19) by differentiation and computing the derivative for the scattering coefficient utilizing a so-called perturbation Monte Carlo.^64^ The approach has been utilized in QPAT for estimation of both absorption and scattering simultaneously.^64^

Due to the stochastic nature of Monte Carlo, the minimization directions evaluated using Monte Carlo simulations are stochastic. Multiple evaluations of Monte Carlo simulation can easily create a bottleneck for the attempts to utilize the method in the solution of the inverse problem. In order to overcome this problem, it was proposed in Ref. 205 to utilize stochastic gradient methods from the machine learning community. In the approach, the number of photon packets utilized in evaluation of the minimization direction is adaptively adjusted based on examining variations of these minimization directions. The approach was utilized in estimation of absorption coefficient using the steepest descent method in Ref. 205, and it was extended to a 2D imaging geometry with a Gauss–Newton algorithm in Ref. 66 and for estimating absorption and scattering simultaneously in Ref. 206.

### 3.3 Single-Stage Approaches

In addition to the two-stage approach, where acoustic and optical inverse problems of QPAT are solved consecutively, estimation of the optical parameters directly from the photoacoustic time-series has been proposed. A single-stage approach was formulated utilizing the Born approximation in Refs. 207 and 208. Furthermore, the $\ell_{1}$ sparsity regularization was utilized,^209^ where the minimization problem was solved with BFGS algorithm also in a limited view measurement geometry, and Tikhonov regularization with a proximal gradient algorithm was utilized in Ref. 177. A single-stage approach for estimating spatially varying speed of sound and optical parameters simultaneously was presented in Ref. 210. A Bayesian approach to determine optical parameters and evaluate the reliability of the estimates was proposed in Ref. 211 and studied also in a limited view measurement geometry and 3D imaging situation. Furthermore, in Ref. 212, stochastic search algorithms were proposed for single-stage QPAT.

## 4 Practical Considerations

This paper is principally concerned with the mathematics of the optical inversions in QPAT, but because inverse problems of this type are inherently motivated by experimental measurements, a brief discussion of the trade-offs and limitations that face experimentalists might help inform future research in this area. The first two subsections below are concerned with the quality and completeness of the acoustic data, which will directly affect the quality of the estimates of optical properties that can be obtained.

### 4.1 Detector Response Versus Sensitivity

Photoacoustic pulses, because of their impulsive nature, are broadband and, because they emanate from a distributed source, may arrive at the detectors from any angle. It was mentioned in Sec. 2.3 that real detectors have the effect of filtering the acoustic pressure both temporally (frequency response) and spatially (directionality). Typically, they are more sensitive over a certain bandwidth (centered at one frequency) and over a range of angles (centered on one angle). There are ways in which the range of frequencies or angles can be increased, but the trade-off is often a drop in the detection sensitivity. As the magnitude of the photoacoustic signals is ultimately limited by safety factors controlling the maximum permissible fluence, signal detection cannot be improved by simply turning up the power. It is often the case, then that photoacoustic measurements are made with detectors whose bandwidths do not capture the full range of available frequencies, and whose directional response does not extend to the steepest angles. Although these responses can be modeled, to some extent, and deconvolved from the data, where the signal has fallen well into the noise it will not be recoverable. The loss of the low frequencies in the data will, without amelioration, lead to a loss of low spatial frequencies in the photoacoustic image, and the loss of the waves arriving from steeper angles can have an effect on the shape or amplitude of the image and lead to “limited-view”-type artifacts.

### 4.2 Array Coverage Versus Cost and Complexity

The ideal detection array would consist of point-like detectors distributed over a surface surrounding the object of interest (spaced at half the shortest acoustic wavelength to satisfy Nyquist), simultaneously streaming data. Practical arrays are prevented from reaching this ideal due to considerations over the cost and complexity of array fabrication, the cost of high-channel count data acquisition systems, and, in some cases, the physical size of the detectors making up the array. Most arrays therefore cover just part of the 2π radians necessary to ensure complete data,^213^ and in some cases, e.g., linear arrays, quite a small fraction, which can lead to “limited-view” artifacts in the image. This hardware limitation can, in some cases, be compensated through scanning or rotating the array to different positions to take new measurements or using compressed sensing strategies, although this increases data acquisition time.

### 4.3 Optical Excitation

How the object is illuminated, both in terms of intensity and coverage, is key to obtaining data with a good signal-to-noise ratio. There are safety limits on the irradiance that soft tissue can be exposed to so illumination over as much surface as is available will maximize the total optical energy entering the tissue. When choosing wavelengths is critical to ensure the well-posedness of the spectroscopic inverse problem, but there is another consideration. Because the depth to which the light will reach depends on the absorbers and scatterers within the tissue, it is not always obvious *a priori* what the optimum optical wavelengths will be in terms of depth penetration, so finding the optimal wavelengths may require trial-and-error. Pulse-to-pulse and wavelength-dependent variations in laser energy must be known but can usually be measured and corrected for. The pulse repetition rate of the laser used affects the data acquisition time (even for systems that use arrays so all the data for one image can be obtained from one pulse, each wavelength requires a different pulse), so the faster the pulses the smaller the risk of tissue motion artifacts in the images. Finally, (this links to the next section) it is important to know how the tissue is being illuminated so that it can be modeled accurately in solving the inverse problem, so the direction (e.g., divergence angle) and intensity of the incident light distribution must be measured or estimated.

### 4.4 Knowledge of Model Parameters

Even when the data are completed, the solution of inverse problem can fail if the auxiliary parameters that are fed into the models are not accurate. Some, such as the positions of the detectors, can be determined through calibration procedures, although this is harder for others, e.g., the positions, directions, and angular spread of the optical sources (mentioned above). Others may be obtainable using another imaging modality, e.g., obtaining the speed of sound using ultrasound tomography. Yet other parameters can be measured on characteristic samples, e.g., the optical anisotropy or Grüneisen parameters, although there will clearly be uncertainties both in these measurements and in how well the samples match the object. In general, all predefined parameters can contain, more or less, uncertainties that will effect on the solution of the inverse problem. Modeling of such uncertainties is one of the research interests in the community of the inverse problems research, and some approaches for tackling the problem Bayesian approximation error modeling^115^ and utilizing machine learning,^135^ have been developed. For example in Ref. 119, it was shown that uncertainties in modeling of ultrasound sensor locations can be compensated using the Bayesian approach.

### 4.5 Computational Considerations

Choosing a solution methodology for an inverse problem is often a trade-off between accuracy of the solution and computational cost. The most simple and straightforward approaches, such as methodologies utilizing analytical solutions of the models, are often limited to specific geometries and/or make unrealistic assumptions of the parameters, assuming scattering as known in the optical inverse problem of QPAT. On the other hand, numerical approximations in large 3D volumes can be computationally expensive, and inverse problems solution methodologies utilizing them require advanced approaches, such as model reduction techniques, to be feasible in practical imaging scenarios. Overall, in practical imaging techniques, it would be desirable that the related modeling and numerical inverse problem methodologies could be implemented such that they could be solved in standard computers without significant time delays. Therefore, although QPAT is a promising methodology for providing high-resolution 3D images of physiologically relevant parameters, there are many computational modeling-based challenges that need to be tackled before the technique can be developed as a standard clinical or preclinical tool.

## 5 Summary

In this survey, the modeling of photoacoustics and inverse problems methodologies for image reconstruction in QPAT were reviewed. As mentioned, QPAT consists of two parts: optical and acoustic, and here we focused on the mathematics of the optical part. Approaches to modeling light propagation in tissue, described by transport theory, and methodologies for the solution of the optical inverse problem were described, but the acoustic aspects of QPAT were discussed only to the extent that they relate to the optical part. Although modeling of light transport is well-understood and there is a well-developed framework of inverse mathematics for approaching the inverse problem of QPAT, there are still challenges in taking these methodologies to practice. For example, although the effect of the variation of the light fluence throughout tissue on photoacoustic tomography images is well-understood, there is no consensus among photoacoustic practitioners on how to remove the effect of the fluence, and thereby facilitate accurate estimation of the tissue’s optical properties.

There are several reasons for this. First, and perhaps most importantly, QPAT is an ill-posed inverse problem. In practice, this means that even small errors in measurements or modeling can cause large errors in the solution of the inverse problem. Related to this is the fact that, while the effect of the optical scattering on the absorbed optical energy density is weaker than that of the absorption, it does not seem to be weak enough to ignore. Second, photoacoustic data are not ideal, as described in Sec. 4, which is a challenging starting point. Third, numerical methods to compute fluence and to solve the inverse problem can be computationally intensive even in simplified imaging situations. Finally, the auxiliary parameters that are needed as inputs to the models (see Fig. 1) can contain uncertainties, making the computational modeling even more difficult. In particular, the Grüneisen parameter may not be known accurately, and methods for compensating its effect on modeling and inverse problem are still being developed.

In this review, we touched these key challenges while giving an overview of computational modeling and inverse problem of QPAT. Furthermore, the developments for tackling these problems were reviewed. A comprehensive literature review was not provided, but we hope that the references can provide a route into broader literature for those who are interested.

## Data Availability

The data that support the findings of this study are available upon reasonable request from the authors.
